# Exploration of Clinical Breakpoint of Danofloxacin for *Glaesserella parasuis* in Plasma and in PELF

**DOI:** 10.3390/antibiotics10070808

**Published:** 2021-07-02

**Authors:** Zihui Xu, Anxiong Huang, Xun Luo, Peng Zhang, Lingli Huang, Xu Wang, Kun Mi, Shiwei Fang, Xiao Huang, Jun Li, Zonghui Yuan, Haihong Hao

**Affiliations:** 1 National Reference Laboratory of Veterinary Drug Residues (HZAU) and MAO Key Laboratory for Detection of Veterinary Drug Residues, Huazhong Agricultural University, Wuhan 430070, China; xuzihui@webmail.hzau.edu.cn (Z.X.); anxionghuang@webmail.hzau.edu.cn (A.H.); luoxun@webmail.hzau.edu.cn (X.L.); cxyzzhangpeng@163.com (P.Z.); huanglingli@mail.hzau.edu.cn (L.H.); wangxu@mail.hzau.edu.cn (X.W.); mikun@webmail.hzau.edu.cn (K.M.); vivifang@mail.hzau.edu.cn (S.F.); HuangAnXiong@163.com (X.H.); lijun@webmail.hzau.edu.cn (J.L.); yuan5802@mail.hzau.edu.cn (Z.Y.); 2 MOA Laboratory for Risk Assessment of Quality and Safety of Livestock and Poultry Products, Huazhong Agricultural University, Wuhan 430070, China

**Keywords:** danofloxacin, *Glaesserella parasuis*, epidemiological cutoff values, PK-PD cutoff values, clinical cutoff values, clinical breakpoint

## Abstract

**Background:** In order to establish the clinical breakpoint (CBP) of danofloxacin against *G. parasuis*, three cutoff values, including epidemiological cutoff value (ECV), pharmacokinetic-pharmacodynamic (PK-PD) cutoff value (CO_PD_) and clinical cutoff value (CO_CL_), were obtained in the present study. **Methods:** The ECV was calculated using ECOFFinder base on the MIC distribution of danfloxacin against 347 *G. parasuis* collected from disease pigs. The CO_PD_ was established based on in vivo and ex vivo PK-PD modeling of danofloxacin both in plasma and pulmonary epithelial lining fluid (PELF) using Hill formula and Monte Carlo analysis. The CO_CL_ was established based on the relationship between the possibility of cure (POC) and MIC in the clinical trials using the “WindoW” approach, nonlinear regression and CART analysis. **Results:** The MIC_50_ and MIC_90_ of danofloxacin against 347 *G. parasuis* were 2 μg/mL and 8 μg/mL, respectively. The ECV value was set to 8 μg/mL using ECOFFinder. Concentration-time curves of danofloxacin were fitted with a two-compartment PK model. The PK parameters of the maximum concentration (C_max_) and area under concentration-time curves (AUC) in PELF were 3.67 ± 0.25 μg/mL and 24.28 ± 2.70 h·μg/mL, higher than those in plasma (0.67 ± 0.01 μg/mL and 4.47 ± 0.51 h·μg/mL). The peak time (T_max_) in plasma was 0.23 ± 0.07 h, shorter than that in PELF (1.61 ± 0.15 h). The CO_PD_ in plasma and PELF were 0.125 μg/mL and 0.5 μg/mL, respectively. The CO_CL_ calculated by WindoW approach, nonlinear regression and CART analysis were 0.125–4 μg/mL, 0.428 μg/mL and 0.56 μg/mL, respectively. The 0.5 μg/mL was selected as eligible CO_CL_. The ECV is much higher than the CO_PD_ and CO_CL_, and the clinical breakpoint based on data in plasma was largely different from that of PELF. **Conclusions:** Our study firstly established three cutoff values of danofloxacin against *G. parasuis.* It suggested that non-wild-type danofloxacin-resistant *G. parasuis* may lead to ineffective treatment by danofloxacin.

## 1. Introduction

*Glaesserella parasuis*, a gram-negative respiratory pathogen, can colonize the upper respiratory tract in swine and cause Glasser’s disease with clinical manifestations such as fibrinous polyserositis, arthritis, meningitis and pneumonia [1]. The serotypes 1, 5, 10, 12, 13 and 14 exhibit higher virulence and pathogenicity [2]. Serotypes 5 and 4 are dominant in China [3]. With the abuse of antibiotics, antimicrobial-resistant *G. parasuis* emerge in different degrees, which bring serious threat to the global economy and public health [4].

Quinolones are effective for treating *G. parasuis* infection because of their strong bactericidal activity and good absorption into the blood and great distribution in the lung [5]. When intravenous injection of danofloxacin 2.5 mg/kg, the distribution half-life was 0.21 ± 0.004 h and Vd area was 6.41 ± 0.0.94 L/kg in sheep plasma [6]. Danofloxacin, one of the most important fluoroquinolones, has a broad spectrum of antimicrobial activity and has been widely used in different animals, such as in sheep [7], bees [8], rabbits [9], turkeys [10], cattle and swine [11]. However, the clinical breakpoint (CBP) for danofloxacin against *G. parasuis* had not yet been established by the clinical laboratory of standard Institute (CLSI) and the European Commission of antimicrobial susceptibility testing (EUCAST).

CBP is set on the basis of epidemiological cutoff values (ECV) or wide-type cutoff (CO_WT_), PK-PD cutoff values (CO_PD_) and clinical cutoff values (CO_CL_) [12]. For a given microbial species and antimicrobial agent, the ECV is the upper bound of the wild-type MIC distribution for organisms with no detectable acquired resistance mechanisms, which can be calculated by nonlinear regression analysis using ECOFFinder software [13,14,15]. CO_PD_ considers the PK-PD parameters of special antimicrobial agents in target animals and uses Monte Carlo simulation to determine the MIC with a 90% possibility of reaching the PK-PD target [16]. CO_CL_ is decided based on the relationship between clinical outcomes and antimicrobial susceptibility using several statistical approaches [17]. The present study aimed to establish the ECV, CO_PD_ and CO_CL_ values for the decision of the final CBP of danofloxacin against *G. parasuis* and evaluation of the efficiency of danofloxacin for treatment of *G. parasuis.*

## 2. Materials and Methods

### 2.1. Strains

From March to May in 2017, a total of 347 *G. parasuis* strains were collected from disease animals. Thirty-five *G. parasuis* strains were isolated from pig lungs provided by Keqian clinical diagnostic center; 8 *G. parasuis* strains were donated by Xiaojuan Xu from State Key Laboratory of Agricultural Microbiology in Huazhong Agricultural University; 204 *G. parasuis* strains were isolated from disease pigs by Peng Zhang in China Agricultural University; 100 *G. parasuis* strains were stored in National Reference Laboratory of Veterinary Drug Residues. All these strains were isolated from the lungs and pericardium of weak or moribund pigs showing respiratory distress or arthritis in different provinces of China. All bacterial isolates were confirmed by PCR amplification of 16S rRNA (Figure S1) [18]. *E. coli* (ATCC 25922) was used as the quality control strain (QC).

### 2.2. Animals

Seventy-eight six-week-old healthy crossbred (Duroc × Large × white × Landrace) pigs weighing 20 ± 2 kg were purchased from Huazhong Agricultural University pig breeding farm. Prior to experiments, pigs were raised for 7 days to acclimatize. Sixteen–twenty g healthy Balb/c mice were purchased from the Experimental Animal Center of Huazhong Agricultural University. Prior to experiments, mice were raised for 7 days to acclimatize. All the animal experiments were approved by the Animal Ethics Committee of Huazhong Agricultural University (hzauch 2014-003) and the Animal Care Center, Hubei Science and Technology Agency in China (SYXK2013-0044). All animal experiments were conducted according to the committee guidelines for the Laboratory Animal Use and Care Committee in Hubei Science and Technology Agency. All efforts were used to reduce the pain and adverse effect of the animals.

### 2.3. Establishment of ECV

Susceptibility testing was performed by agar dilution method according to CLSI M07-A9 standard with some modification [19]. A 2 μL *G. parasuis* suspension (10^7^ CFU/mL measured by Mcfarland Turbidimetric Method) was inoculated onto TSA-FCS-NAD agar plates containing two-fold dilutions (0.008, 64 μg/mL) of danofloxacin (Dr. Ehrenstorfer Standards, Augsburg, Germany). The MICs were converted to Log scale, ECV was simulated using ECOFFinder software [20]. ECV at 95%, 97.5%, 99%, 99.5% and 99% confidence intervals were simulated. Generally, the ECV with the 95% confidence interval is selected as the final ECV.

### 2.4. Establishment of CO_PD_ Based on PK-PD Modeling

#### 2.4.1. Selection of Pathogenic *G. parasuis*

The serotype of 81 strains whose MIC was the same as MIC_90_ and higher than MIC_90_ were determined by ERIC-PCR (Enterobacterial Repetitive Intergenic Consensus - PCR) using ERIC primer (5′-ATG TAA GCT CCT GGG GAT TCA C-3′ and 5′-AAG TAA GTG ACT GGG GTG AGC G-3′) following previous study (Figure S2) [21,22]. SH 0165 (serotype 5) was the positive control.

The 18 strains of serotype 5 were selected for the mouse pathogenicity test. The 16–20 g healthy Balb/c mice were randomly divided into 19 groups (5 mice/group) with one blank control group. The mice were inoculated with 1 × 10^9^ cfu bacteria by abdominal cavity injection, and the control group was injected with TSB broth. Mice were monitored daily for 7 days post-inoculation (dpi). The pathogenicity of *G. parasuis* was compared according to the survival time [23].

#### 2.4.2. Pharmacodynamics In Vitro and Ex-Vivo

The MIC and MBC of *G. parasuis* H80 in broth and pulmonary epithelial lining fluid (PELF) were determined using the broth dilution method according to the CLSI M07-A9 standard with some modification.

The in vitro and ex vivo killing curves of danofloxacin in broth and in PELF were drawn by monitoring the Colony formed unite (CFU) changes during the incubation of *G. parasuis* H80 under a series concentration of danofloxacin (1/2 to 32 MIC) for a continuous time period (0, 1, 2, 4, 6, 8, 12 and 24 h).

#### 2.4.3. Animal Experiment and Sample Collection for Pharmacokinetics Study

Danofloxacin was administrated to twelve pigs at a single dose of 2.5 mg/kg b.w. by intramuscular injection. After administration, 2 mL blood samples were obtained at 0, 0.08, 0.17, 0.25, 0.5, 0.75, 1, 1.5, 2, 3, 4, 6, 8, 10, 12, 24, 36 and 48 h. Plasma was obtained by centrifuging the blood sample at 3500 r/min for 10 min, and the sample was stored at −80 °C before processing.

To collect PELF samples, atropine (0.05 mg/kg) and propofol (9–15 mg/kg) were given intramuscularly and intravenously for 30 min for anesthesia. Standardized Bronchoalveolar Lavage (BAL) was performed as previously described [24,25], with an electronic fiber optic bronchoscope (KangmeiGU-180VET) inserted in the right middle lung lobe. The 50 mL of normal saline was instilled into the lobe and was aspirated into a 50 mL centrifugal tube. The PELF samples were collected at 0, 0.5, 1, 1.5, 2, 4, 6, 8, 10, 12, 24, 36 and 48 h. The PELF was centrifuged at 800 r/min for 10 min, and the sample was stored at −80 °C before processing.

#### 2.4.4. Quantitation Analysis of Danofloxacin by HPLC

Quantitation analyses of danofloxacin in PELF and plasma were conducted using high-performance liquid chromatography (HPLC). Agent SB-Aq reverse-phase column (250 mm, 4.6 mm i.d., 5 mm; Agilent) was used to perform HPLC at 30 °C. The detection wavelength was 280 nm. The mobile phase consisted of 0.05% phosphoric acid (phase A) and acetonitrile (phase B) with gradient elute. The peak time of danofloxacin was 10.64 min. 0.5 mL Plasma and 0.5 mL PELF were extracted with 2 mL acetonitrile twice.

The urea dilution method was used to determine the volume of PELF as described previously [26,27]. The concentration of urea in plasma (Urea_PLASMA_) and PELF (Urea_PELF_) were determined by using a urea test kit (Urea test kit; Sigma Chemical, St. Louis, MO, USA) and the absorbance values measured by using a spectrophotometer (Agilent 8453, Wuhan, China). The final concentration of danofloxacin in PELF (C_PELF_) was derived from the following equation: $C_{\mathrm{PELF}}=C_{\mathrm{BAL}}\times \left(\frac{{\mathrm{Urea}}_{\mathrm{PLASMA}}}{{\mathrm{Urea}}_{\mathrm{PELF}}}\right)$; C_BAL_ was the diluted concentration of danofloxacin in PELF determined by the HPLC method.

#### 2.4.5. Pharmacokinetics-Pharmacodynamics Modeling

PK-PD parameters were estimated using Winnonlin (v.5.2.1 US Certara Pharsight^®^) with a two-compartment model. According to the ex vivo time-killing curve, the Sigmoid E_max_ model ($E=E_{0}-\frac{{\mathrm{PD}}_{\max}\times X^{\gamma }}{X^{\gamma }+{\mathrm{EC}}_{50}^{\gamma }}$) was used to calculate the AUC_24_/MIC (AUIC) of danofloxacin at different concentrations, E is the summary PD endpoint, and E_0_ is the effect representing the value of the PD endpoint without drug treatment (i.e., the value of the summary endpoint when the PK-PD index is 0). X is one of the three PK-PD indices as defined above, and PD_max_ is the maximum effect (in relation to E_0_) indicated by the plateau where increased exposures result in no further kill. EC_50_ is the magnitude of X that is needed to achieve 50% of PD_max_, and γ is the sigmoidicity factor. The PD target under different efficiency (E = 0, −3 and −4 (bacteriostasis, bactericidal and eradication)) was determined with *Sigmoid E_max_* equation [28,29]. The dosage regimen was derived from the concentration-dependent dosage equation ($\mathrm{Dose}=\frac{\mathrm{MIC}\times \mathrm{AUIC}}{\mathrm{fu}}\times \mathrm{CL}/F$) [30,31,32]. In the equation, the CL (mL/h) was the plasma (total) clearance per day, AUIC (h) was the targeted endpoint for optimal efficacy, fu was the free fraction of the drug in PELF (from 0 to 1), and F was the bioavailability factor (from 0 to 1). In this study, fu was 0.8974, which was obtained by measuring the protein binding rate by the equilibrium dialysis method.

#### 2.4.6. Monte Carlo Simulation to Set up CO_PD_

Crystal Ball v7.2.2 was used to perform the Monte Carlo simulation. The distribution of the PK-PD parameter was assumed to be log-normal. A total of 10,000 subjects were simulated. The PD target was selected to calculate the probability of target attainment (PTA). CO_PD_ was defined as the MIC at which the PTA was ≥90%.

### 2.5. Clinical Trial and Establishment of CO_CL_

#### 2.5.1. Infection Model and Clinical Trials

Sixty-six healthy weaned piglets (20 ± 2 kg) were divided into 11 groups: 5 groups were the experimental group, 5 groups were the negative control group, and 1 group was the blank control group, with 6 piglets in each group. The 5 experimental groups and 5 negative control groups were challenged with 5 representative strains, H42, H80, H12, H83 and H17, by intranasal inoculation of 1 × 10^10^ CFU bacterial suspension twice a day. The blank control group was inoculated with blank TSB broth. The dosage regimens were recommended by the PK-PD therapeutic dosage regimen. After challenging, these pigs were monitored daily for two weeks.

#### 2.5.2. Statistical Analysis for Establishment of CO_CL_

The probability of cure (POC) was calculated based on the clinical outcomes and bacteriological prognosis. Clinical outcomes included treatment success and failure, and each MIC should have a corresponding clinical outcome. The bacteriological prognosis was to determine the presence or eradication of the bacteria after administration. The data were analyzed by three different analysis methods.

The “WindoW” approach [17] included two parameters: “MaxDiff” and “CAR”. “MaxDiff (the method of maximum difference, MaxDiff)” represents the difference between higher and lower POC at a certain MIC. “CAR” was based on the clinical outcome and the corresponding MIC distribution. “CAR” could not be set as the lowest MIC or the highest MIC if “CAR” was gradually increasing with MIC, then the “CAR” should choose the second small “CAR”.

Nonlinear regression analysis was a new method based on the formula between EUCAST proposed POC with MIC. Log_2_MIC was the independent variable, and the POC was the dependent variable. The model with the highest correlation coefficient was selected to simulate its CO_CL_.

The classification and regression tree (CART) model (Salford Predictive Modeler software) was also used for the establishment of CO_CL_. MIC was used as the predictive variable, and the POC was the target variable. The Gini coefficient minimization criterion was used to select the MIC node automatically.

## 3. Results

### 3.1. ECV for Danofloxacin against G. parasuis

**Figure 1:**
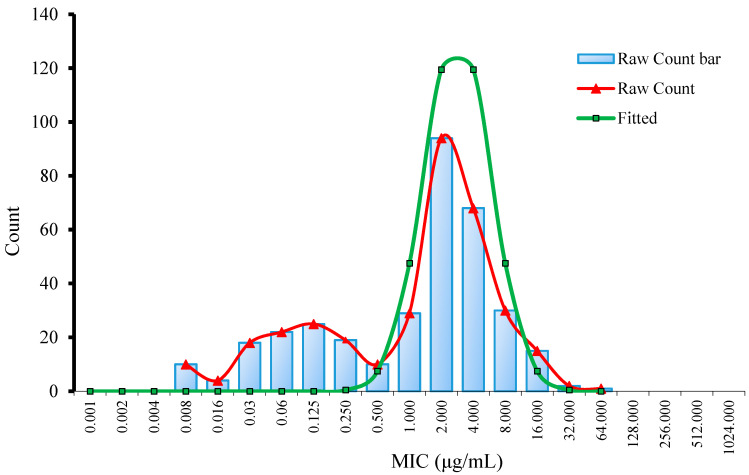
Nonlinear regression of MIC distribution for danofloxacin against *G. parasuis*.

The MIC distribution for danofloxacin against *G. parasuis* is shown in Figure 1. The MIC of danofloxacin ranged from 0.008 to 64 μg/mL. As shown in Figure 1, the MIC distribution was as follows: 0.008 µg/mL (2.88%), 0.015 µg/mL (1.15%), 0.03 µg/mL (5.19%), 0.06 µg/mL (6.34%), 0.125 µg/mL (7.20%), 0.25 µg/mL (5.48%), 0.5 µg/mL (2.88%), 1 µg/mL (8.36%), 2 µg/mL (27.09%), 4 µg/mL (19.60%), 8 µg/mL (8.65%), 16 µg/mL (4.33%), 32 µg/mL (0.58%) and 64 µg/mL (0.29%). The MIC_50_ and MIC_90_ were 2 μg/mL and 8 μg/mL, respectively.

Using the ECOFFinder software, the fitted MIC distribution of danofloxacin against *G. parasuis* was shown in Figure 1. The ECV at 95%, 97.5%, 99%, 99.5% and 99.9% confidence intervals were 8, 8, 16, 16 and 32 μg/mL, respectively (Table S1).

### 3.2. CO_PD_ for Danofloxacin against G. parasuis

#### 3.2.1. Pathogenic *G. parasuis*

Eighteen strains belonging to serotype 5 were selected from ERIC-PCR amplification and pathogenicity test in mice, and five strains (H42, H80, H12, H83 and H17) showed the highest pathogenicity and exhibited different MIC. The strain H80 with MIC close to MIC_50_ was selected for the PK-PD study. The five respective strains H42 (MIC = 16 µg/mL), H80 (MIC = 4 µg/mL), H12 (MIC = 1 µg/mL), H83 (MIC = 0.125 µg/mL) and H17 (MIC = 0.015 µg/mL) were selected for clinical trial.

#### 3.2.2. Pharmacodynamics of Danofloxacin against *G. parasuis*

The MICs of danofloxacin in broth and pulmonary epithelial lining fluid (PELF) were 4 μg/mL and 2 μg/mL, respectively. The MBC in broth and PELF were 8 μg/mL and 4 μg/mL, respectively. The antibacterial activity of danofloxacin in PELF is stronger than that of in broth. 

**Figure 2:**
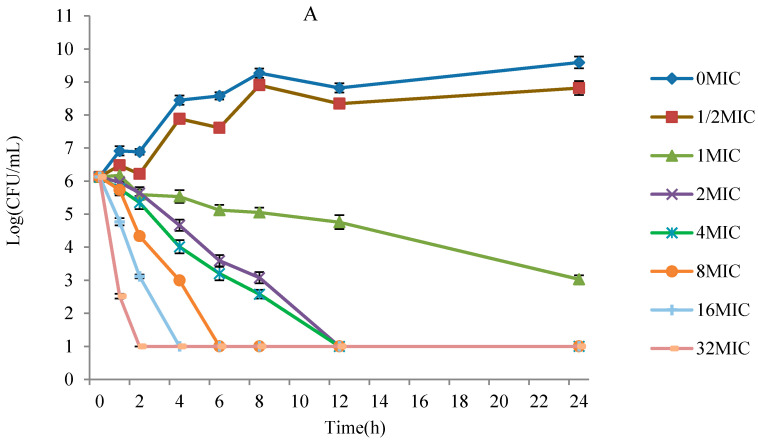

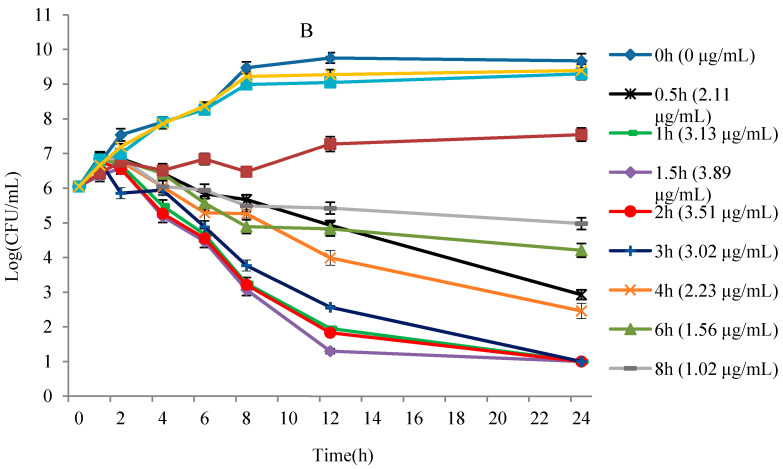
The killing curve of *G. parasuis* in PELF and plasma. (**A**) is the killing curve of *G. parasuis* in TSB broth, and (**B**) is the killing curve of *G. parasuis* in PELF.

As displayed in Figure 2, the in vitro and ex vivo bactericidal effect of danofloxacin against *G. parasuis* was similar. The lower concentrations (≤MIC) of danofloxacin exhibited similar antibacterial activity to *G. parasuis*. However, when danofloxacin concentrations were higher than MIC, the inhibitory efficiency gradually strengthened following the increased drug concentration. The time-killing curve showed that the activity of danofloxacin against *G. parasuis* was concentration-dependent. The Aera Under Curve/Minimum Inhibitory Concentration (AUC/MIC) was selected as the PK-PD parameter.

#### 3.2.3. Sensitivity and Accuracy of HPLC Method for Determination of Danofloxacin

The limit of determination (LOD) was 0.01 μg/mL, and the limit of quantification (LOQ) was 0.025 μg/mL in PELF. The LOD was 0.02 μg/mL, and the LOQ was 0.05 μg/mL in plasma. Standard curves were linear from 0.05 μg/mL to 5 μg/mL in plasma (R^2^ = 0.9994) and 0.025 μg/mL to 2.5 μg/mL in PELF (R^2^ = 0.9996). The inter-day variation for determination in plasma and PELF ranged from 1.94% to 2.37% and 1.36% to 2.71%, respectively. The recovery of danofloxacin in plasma and PELF ranged from 90.79 ± 2.15 to 94.36 ± 1.83 and 91.91 ± 2.49 to 95.73 ± 1.30, respectively.

#### 3.2.4. PK Characteristics of Danofloxacin in Plasma and PELF

**Figure 3:**
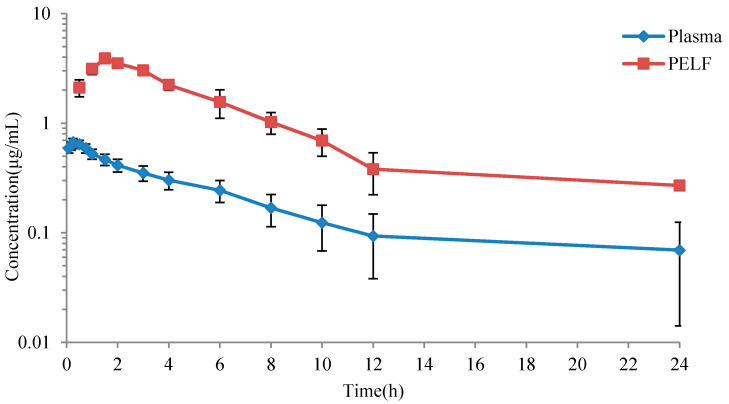
The mean concentration versus time curves for danofloxacin in PELF and plasma.

The concentration-time curves in plasma and PELF after administration of danofloxacin at a single dose of 2.5 mg/kg b.w. are shown in Figure 3. Concentrations of danfloxacin in plasma and PELF at various time points are shown in Table S2. A striking difference is observed between drug concentrations in plasma and in PELF.

The estimated pharmacokinetic parameters in plasma and PELF were shown in Table 1. Distribution of danofloxacin in simulated drug time curve in plasma and in PELF were shown in Figures S3 and S4. In plasma, the peak time (T_max_) was 0.23 ± 0.07 h, the peak concentration (C_max_) was 0.67 ± 0.01 μg/mL, the area under the concentration-time curves (AUC) was 4.47 ± 0.51 h·μg/mL; in PELF, T_max_ was 1.61 ± 0.15 h, C_max_ was 3.67 ± 0.25 μg/mL, AUC was 24.28 ± 2.70 h·μg/mL.

Combined with the killing curve in PELF, the PD target (AUIC in ex vivo) under different efficiency was calculated by Sigmoid E_max_ equation simulation (Table 2). The values of AUIC (h) at E = 0, −3 and −4 (bacteriostasis, bactericidal and eradication) were 12.73, 28.68 and 44.38, respectively.

#### 3.2.5. Monte Carlo Simulation and CO_PD_

According to the AUC (24.28 ± 2.70 h·μg/mL) and PD target (12.73, 28.68, 44.38) in PELF, Monte Carlo analysis simulated the possibility of target achievement (PTA) under different MICs (Table 3 and Figure S5). When the PTA in PELF was upon 90%, the CO_PD_ (E = 0, −3, −4) for danofloxacin against *G. parasuis* in PELF was 1 μg/mL, 0.5 μg/mL, 0.25 μg/mL, respectively.

According to the AUC (4.47 ± 0.51 h·μg/mL) and PD target (12.73, 28.68 and 44.38) in plasma, Monte Carlo analysis simulated the PTA under different MICs (Table 3 and Figure S6). When the PTA in plasma was upon 90%, the CO_PD_ (E = 0, −3, −4) for danofloxacin against *G. parasuis* in plasma was 0.25 μg/mL, 0.125 μg/mL and 0.03 μg/mL, respectively.

### 3.3. CO_CL_ of Danofloxacin against G. parasuis

The dosage under different efficiency (bacteriostasis, bactericidal and eradication) were 4.58 mg/kg, 10.32 mg/kg and 15.97 mg/kg. The given dosages were simulated by Mlxplore software (Figure S7). The modified dosage regimen was 12.49 mg/kg danofloxacin twice a day. Three methods were used to obtain CO_CL_ according to the relationship between POC and MIC distribution (Table 4).

Following the “WindoW” method, the parameters of MaxDiff (0.28) and CAR (0.78) was corresponding with the MIC of 0.125 μg/mL and 4 μg/mL, respectively. Therefore, the CO_CL_ selection window range is 0.125 μg/mL to 4 μg/mL. The nonlinear regression model was set up as $y=80.989-7.271x+0.271x^{2}+0.16x^{3}$ with a correlation coefficient of 0.996. When POC was 90%, the recommended CO_CL_ (MIC) was less than 0.428 μg/mL. The CART regression tree indicated that the CO_CL_ was less than 0.56 μg/mL (Figure S8). Combined with the above three results, the CO_CL_ of danofloxacin against *G. parasuis* was selected as 0.25 μg/mL.

## 4. Discussion

*G. parasuis* is an important respiratory pathogen in swine. Antimicrobial treatment is the more effective way to control this pathogen due to vaccine deficiency. However, antimicrobial resistance in *G. parasuis* had been found in Germany [33], the United Kingdom, Spain [34] and China [35,36,37]. In order to rationally use antimicrobials agents to control *G. parasuis*, some studies have been conducted to establish the ECVs and/or CO_PD_ of marbofloxacin, cefquinome and tilmicosin against *G. parasuis* [29,38,39]. Danofloxacin is very effective against *Actinobacillus pleuropneumoniae* [40], *Pasteurella multocida* [41] and *Mannheimia haemolytica* [42]. However, the clinical breakpoint of danofloxacin against *G. parasuis* had not yet been established.

Statistical analysis had been widely used for the determination of ECVs. Turnidge [13] recommends using nonlinear regression to analyze the obtained MIC data and determined the ECVs of various drugs. Kronvall [43] used NRI (Normalized Resistance Interpretation) method to analyze MIC data obtained by E test for the establishment of ECVs. European Commission of Antimicrobial Susceptibility Testing (EUCAST) recommended ECOFFinder software on the basis of Turnidge’s nonlinear regression [44]. Van Vliet [45] used NRI and ECOFFinder analysis method to analyze wild-type cutoff values of ampicillin, florfenicol, gentamicin and enrofloxacin. In our study, the ECV of danofloxacin determined by nonlinear regression analysis was the same as that simulated by ECOFFinder software, suggesting that ECOFFinder software is a convenient tool for the establishment of ECVs. In the present study, the MIC distribution of danofloxacin against *G. parasuis* appeared three peaks (0.008 μg/mL, 0.125 μg/mL and 2 μg/mL), suggesting that some *G. parasuis* isolates may be resistant to danofloxacin. Zhang et al. [46] examined the resistance of 138 *G. parasuis* strains against fluoroquinolone drugs and showed that 60.1% of isolates were resistant to enrofloxacin, and 5.8% of isolates were resistant to levofloxacin. It suggested that *G. parasuis* may also be resistant to danofloxacin due to the cross-resistance between fluoroquinolone drugs.

The CO_PD_ was established based on pharmacokinetic data, MIC distribution and PK-PD target. Our present study establishes the CO_PD_ based on the PK data from healthy animals because of the stability and repeatability of a healthy animal model. Considering the drug concentrations in the target sites were directly correlated with clinical efficacy, the PK data both in plasma and in PELF were included in our study [47]. Similar to previous studies, our results indicated that the concentration and AUC of danofloxacin in PELF (in the lung) was 4–7 times higher than that in plasma [11]. The CO_PD_ of danofloxacin in PELF was subsequently higher than the CO_PD_ in plasma, indicating that the CO_PD_ was different between in the target tissue and in plasma. As danofloxacin can be accumulated at the infection site (lung), the CO_PD_ in plasma may not represent the critical value of the target tissue. It was of great significance to establish the CO_PD_ in target tissue and plasma simultaneously. The differences in pharmacokinetic parameters between different studies may be due to differences in pig breeds or individuals. In this study, the T_max_ of pigs after i.m. administration of danofloxacin at a dose of 2.5 mg/kg b.w. was 0.23 ± 0.07 h, and this result is different from the result reported by Yang [48] at 0.97 ± 0.08 h; C_max_ was 0.67 ± 0.01 μg/mL, which is in good agreement with the previously reported 0.76 ± 0.08 μg/mL; the AUC_24h_ was 4.47 ± 0.51 h·μg/mL, which is less than 5.25 ± 1.35 h·μg/mL, as reported by Yang et al.

Previously, a study exhibited good clinical outcomes of danofloxacin in the treatment of respiratory disease caused by *Haemophilus somnus* and *Pasteurella multocida* in European cattle [49]. The clinical data in our study also showed the good clinical outcome of danofloxacin in the treatment of *G. parasuis* in pigs because the success rate for treatment of *G. parasuis* with MIC of 1 μg/mL was still as high as 83.33%. The CO_CL_ was established based on the relationship between MIC and POC under modified therapeutic dosage. Since there was no standard approach for the establishment of CO_CL_, the CO_CL_ in the present study was established by the combination of the three approaches, which included the “WindoW” approach [17], the nonlinear regression [50] and the CART analysis [51,52]. The “WindoW” approach was recommended by CLSI [17]. The nonlinear regression with the formula of POC = 1/(1 + e^-a+bf (MIC)^) was proposed by VetCAST to calculate the relation between the dependent variable of POC and the independent variable of MIC [50]. The CART method was previously used to develop clinical breakpoints of cefepime [53], and this method was recommended by Dr. Cuesta [54] and Prof. Toutain [12] because the CART obtained the best statistical results when it was compared with other four supervised classifiers (J48, the OneR decision rule, the naïve Bayes classifier and simple logistic regression).

A large difference was observed between three cutoff values with ECV higher than CO_PD_ and CO_CL_. In previous studies, there was data that showed the MIC breakpoint of danofloxacin against *Mannheimia haemolytica* and *Pasteurella multocida* was 1 μg/mL [55], while Yang’s data showed that the epidemiologic cutoff value of danofloxacin against *E. coli* was 8 μg/mL [48], which was in accordance with our study. The difference of ECV between different studies may be due to the epidemiological characteristic of a different bacterial in different geography. Additionally, previous data showed that some of *G. parasuis* isolates exhibited decreased sensitivity to fluoroquinolones [56]. Three peaks of MIC distribution in the present data also suggested that some *G. parasuis* isolates may be resistant to danofloxacin. The higher MIC of the resistant isolates may contribute to the higher ECV value, and further studies may need to confirm the relationship between MIC phenotype and resistance genotype.

## 5. Conclusions

This study firstly established the ECV (8 μg/mL) at 95% confidence intervals, CO_PD_ in PELF (0.5 μg/mL), CO_PD_ in plasma (0.125 μg/mL) and CO_CL_ (0.25 μg/mL) of danofloxacin against *G. parasuis*. Based on the CLSI decision tree, the final CBP in plasma and PELF was 0.25 μg/mL and 8 μg/mL, respectively (Figure S9). The ECV value was higher than CO_PD_ and CO_CL_, indicating that some *G. parasuis* isolates may be resistant to danofloxacin.

## Figures and Tables

**Table 1:** PK parameters of danofloxacin in plasma and PELF (*n* = 6).

Parameters	Unit	Plasma	PELF
A	μg/mL	0.43 ± 0.16	6.50 ± 2.21
B	μg/mL	0.37 ± 0.18	0.54 ± 0.40
α	1/h	0.40 ± 0.13	0.29 ± 0.04
β	1/h	0.14 ± 0.02	0.06 ± 0.02
K01	1/h	25.04 ± 32.21	1.41 ± 0.50
K10	1/h	0.13 ± 0.03	0.10 ± 0.85
K12	1/h	0.12 ± 0.04	0.17 ± 0.78
K21	1/h	0.13 ± 0.10	0.02 ± 0.18
T_1/2K01_	h	0.03 ± 0.03	0.49 ± 0.17
T_1/2α_	h	1.78 ± 0.76	2.39 ± 0.3
T_1/2β_	h	4.96 ± 0.47	10.46 ± 0.76
T_max_	h	0.23 ± 0.07	1.61 ± 0.15
AUC_24_	h·μg/mL	4.47 ± 0.51	24.28 ± 2.70
C_max_	μg/mL	0.67 ± 0.01	3.67 ± 0.25
CL/F	mL/h/kg	571.49 ± 53.02	89.98 ± 9.7
Vd/F	mL/kg	3531.73 ± 49.12	435.04 ± 45.43

A and B: Y-axis intercept terms; α and β: exponential coefficients; K01: absorption rate constant; K10: central compartment elimination rate constant; K12: distribution rate constant from a central to a peripheral compartment; K21: distribution rate constant from a peripheral to a central compartment; T_1/2K01_: absorption half-life of the drug; T_1/2α_: half-life of α phase; T_1/2β_: half-life of β phase; T_max_: the time point of maximum plasma concentration of the drug; AUC: area under the curve of plasma concentration-time; C_max_: the maximum plasma concentration; CL/F: the apparent volume of the central compartment cleared of drug per unit time; Vd/F: Apparent volume of distribution based on the terminal elimination phase.

**Table 2:** The PD target of danofloxacin against *G. parasuis*.

Time (h)	C_vivo_	(AUIC)_ex_	E (logCFU/mL)	Calculated PD Target
0	0.00	0.00	3.62	E_0_ = 3.62 PD_max_ = 8.67 EC_50_ = 15.24 γ = 1.85 AUIC (*E* = 0) = 12.73 AUIC (*E* = −3) = 28.68 AUIC (*E* = −4) = 44.38
0.5	2.11 ± 0.37	25.34 ± 4.39	−3.12	
1	3.13 ± 0.35	37.54 ± 4.21	−5.05	
1.5	3.89 ± 0.11	46.70 ± 1.37	−5.05	
2	3.51 ± 0.33	42.15 ± 3.96	−5.05	
3	3.02 ± 0.21	36.28 ± 2.53	−5.05	
4	2.23 ± 0.25	26.81 ± 2.95	−3.59	
6	1.56 ± 0.45	18.72 ± 5.39	−1.84	
8	1.02 ± 0.23	12.28 ± 2.75	−1.07	
10	0.69 ± 0.19	8.31 ± 2.33	1.49	
12	0.38 ± 0.16	4.56 ± 1.90	3.24	
24	0.27 ± 0.03	3.24 ± 0.31	3.34	

C_vivo_ is the concentration of danofloxacin in PELF; (AUIC)_ex_ is selected PK-PD parameters; a represented the bacterial colonies lower than the limit of detection (10 CFU/mL).

**Table 3:** The PTA of danofloxacin against *G. parasuis* at different MICs in PELF and plasma.

MIC (μg/mL)	PELF			Plasma		
	PTA% (E = 0)	PTA% (E = −3)	PTA% (E = −4)	PTA% (E = 0)	PTA% (E = −3)	PTA% (E = −4)
0.015	100	100	100	100	100	100
0.03	100	100	100	100	100	100
0.125	100	100	100	100	98.46	1.24
0.25	100	100	100	99.94	0	0
0.5	100	100	80.97	0.04	0	0
1	100	3.81	0	0	0	0
2	29.95	0	0	0	0	0
4	0	0	0	0	0	0
8	0	0	0	0	0	0
16	0	0	0	0	0	0
32	0	0	0	0	0	0

**Table 4:** POC and “WindoW” for danofloxacin against *G. parasuis* at different MIC.

Strain Number	Strain Group	MIC (μg/mL)	Success (%)	Eradication (%)	POC (%)	MaxDiff	CAR
H42	Test	16	67.7	67.7	67.7	0	0.70
	Control		16.7	0	0		
H80	Test	4	67.7	83.3	67.7	0	0.79
	Control		33.3	16.7	33.3		
H12	Test	1	83.3	83.3	83.3	0.167	0.93
	Control		33.3	16.7	33.3		
H83	Test	0.125	100	100	100	0.28	1
	Control		33.3	16.7	16.7		
H17	Test	0.015	100	100	100	0.21	1
	Control		50	33.3	33.3		

## Data Availability

Data is contained within the article.

## Supplementary Materials

The following are available online at https://www.mdpi.com/article/10.3390/antibiotics10070808/s1, Figure S1: Amplification of G. parasuis 16S rRNA with PCR, Figure S2: Results of ERIC-PCR for *G. parasuis*, Figure S3: Distribution of danofloxacin in simulated drug time curve in plasma, Figure S4: Distribution of danofloxacin in simulated drug time curve in PELF, Figure S5: PTA of danofloxacin against *G. parasuis* in PELF, Figure S6: PTA of danofloxacin against *G. parasuis* in plasma, Figure S7: Forecast growth of *G. parasuis* at different dosage regimens, Figure S8: CART tree showing values of clinical outcome, Figure S9: Susceptibility breakpoint decision tree, Table S1: Epidemiological MIC for danofloxacin against *G. parasuis*, Table S2: Concentrations of danfloxacin in plasma and PELF at various time points (*n* = 6).
