# Supplementary material for: Exploration of Clinical Breakpoint of Danofloxacin for Glaesserella parasuis in Plasma and in PELF
Source: Antibiotics (Basel). 2021 Jul 2;10(7):808. doi: 10.3390/antibiotics10070808 (PMC8300709; doi:10.3390/antibiotics10070808)
Supplement: Supplementary file 1 [file antibiotics-10-00808-s001.zip › antibiotics-1223837-supplementary.pdf]

antibiotics

MDPI

Supplementary materials

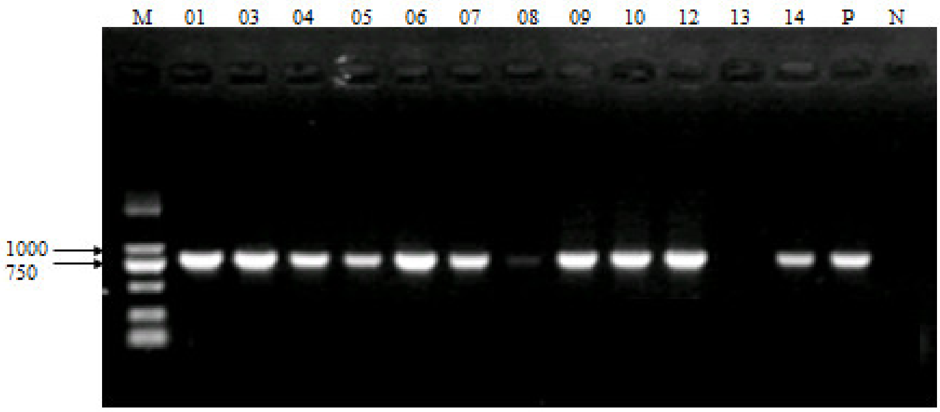

Figure S1. Amplification of *G. parasuis* 16S rRNA with PCR. Lane M: DL-2000 DNA Marker; Lane P: positive control; Lane N: Negative control; Lane2-13: Samples to be amplificated.

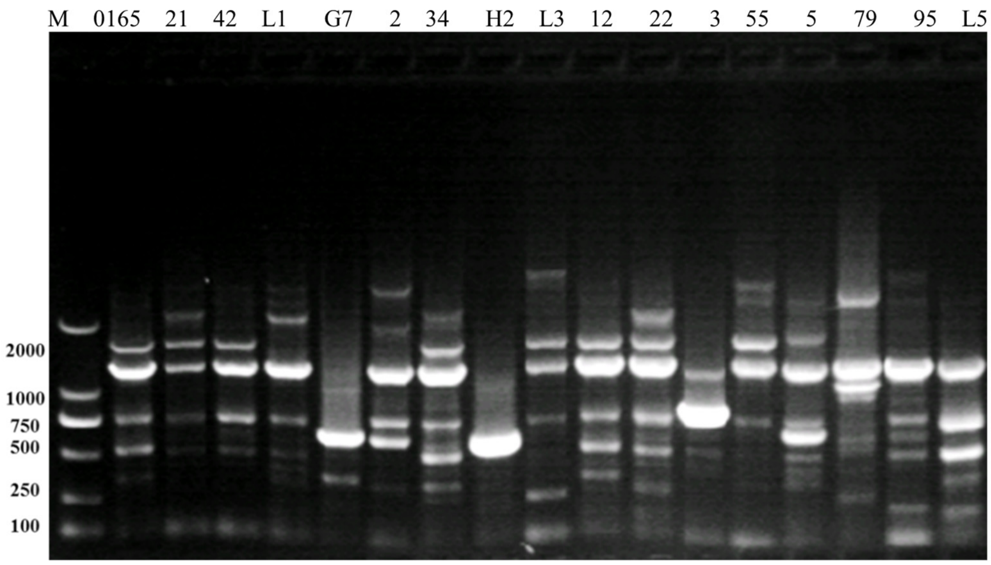

Figure S2. Results of ERIC-PCR for *G. parasuis*. Lane M: DL-2000 DNA Marker; Lane 2: SH0165 strain; Lane 17: Negative control; Lane 3-16: Samples to be amplificated.

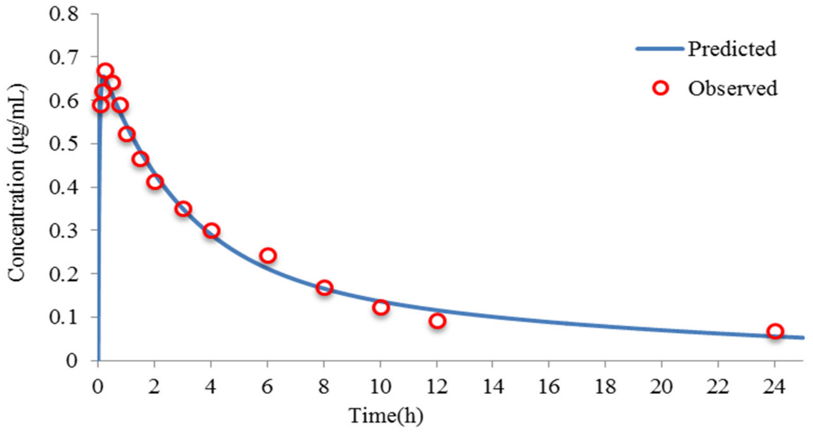

Figure S3. Distribution of danofloxacin in simulated drug time curve in plasma.

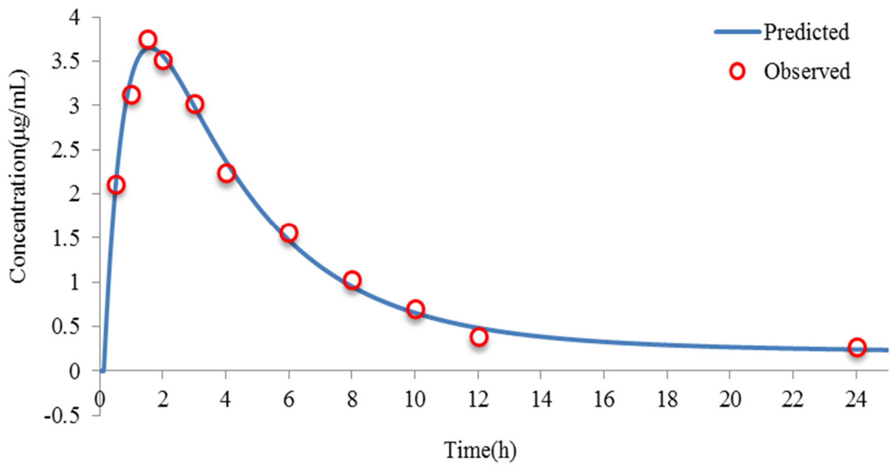

Figure S4. Distribution of danofloxacin in simulated drug time curve in PELF.

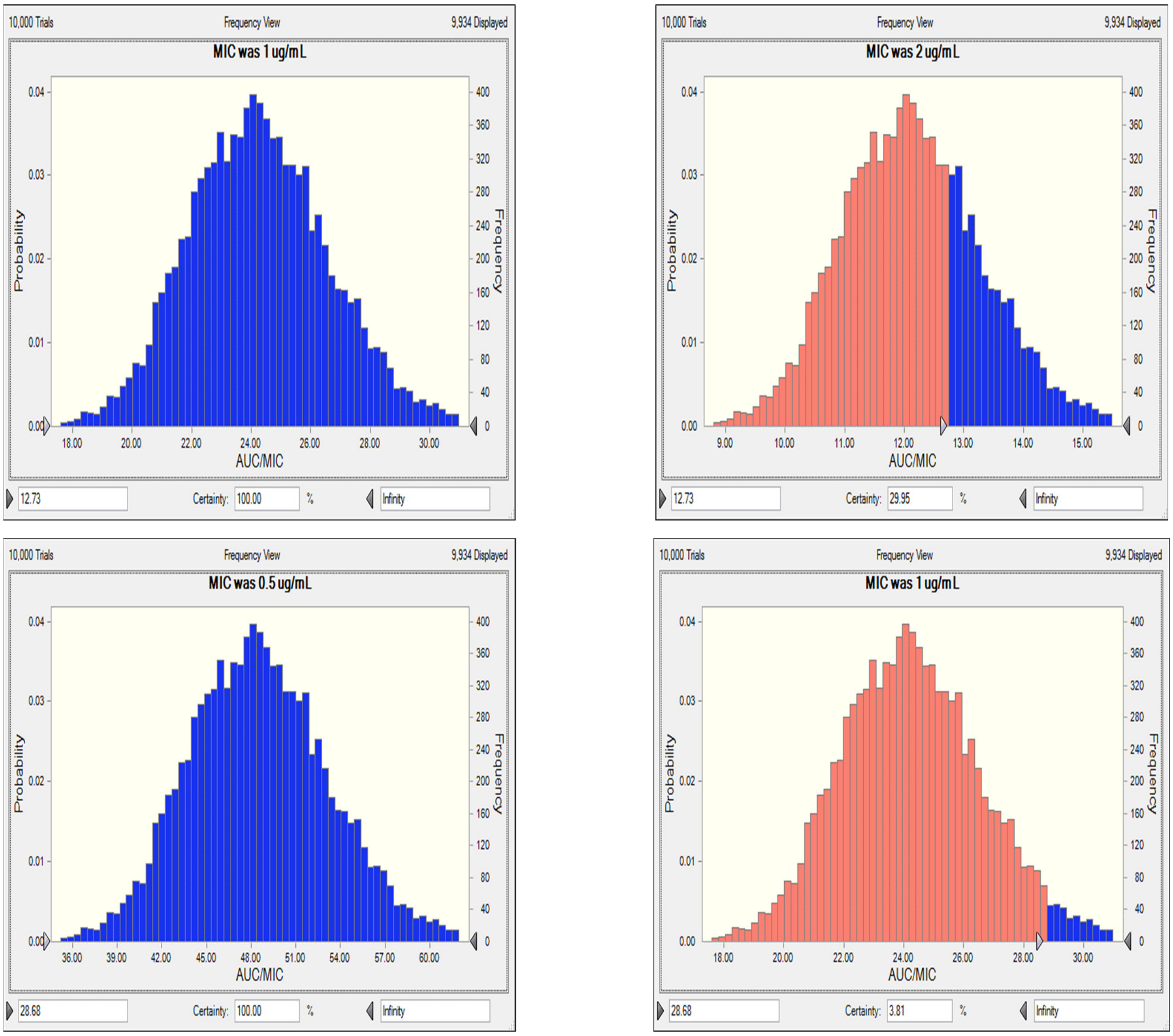

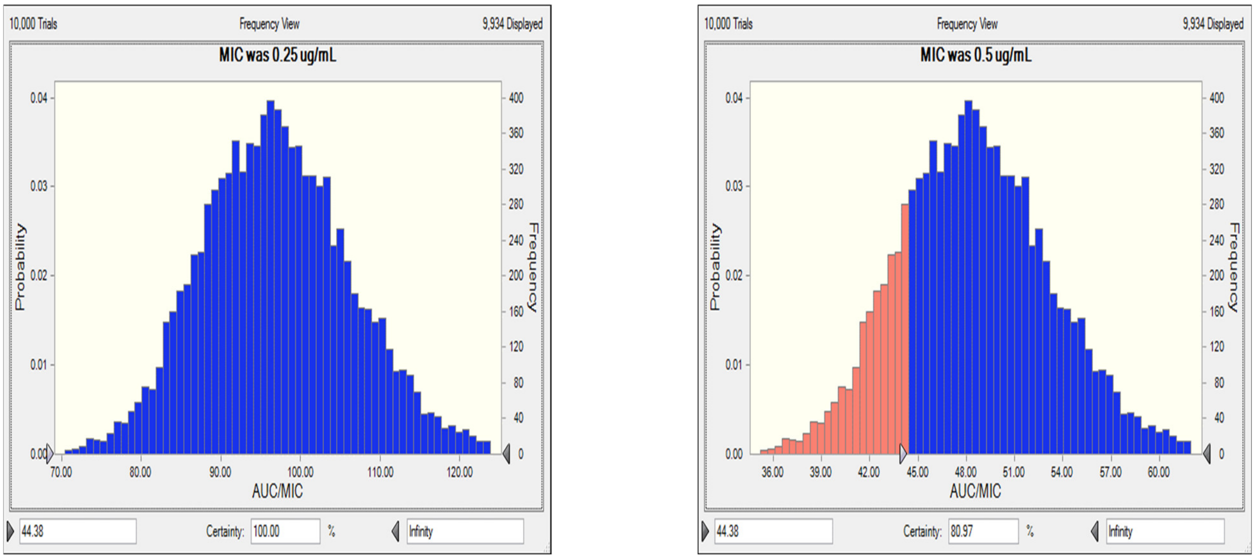

Figure S5. PTA of danofloxacin against *G. parasuis* in PELF.

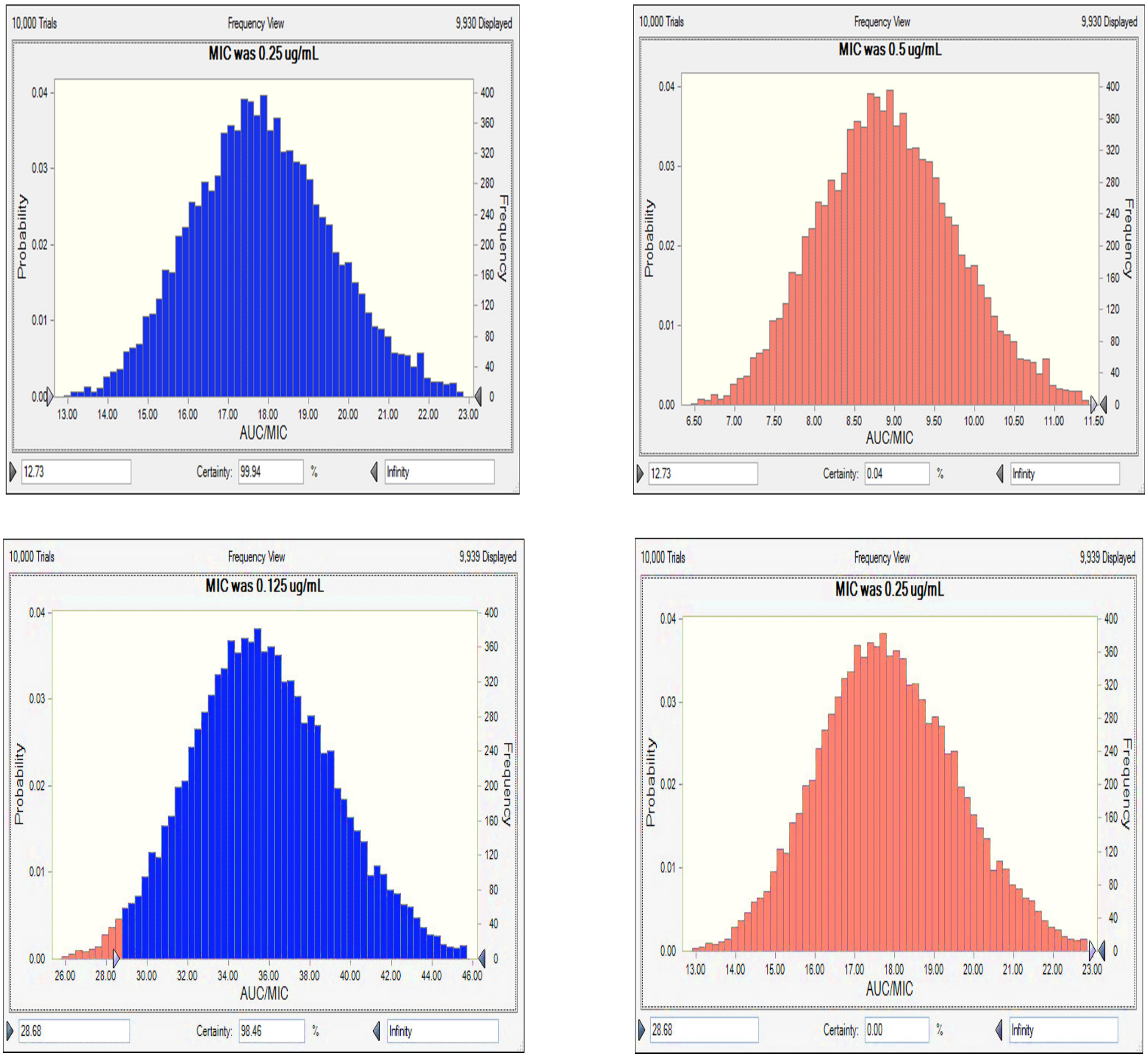

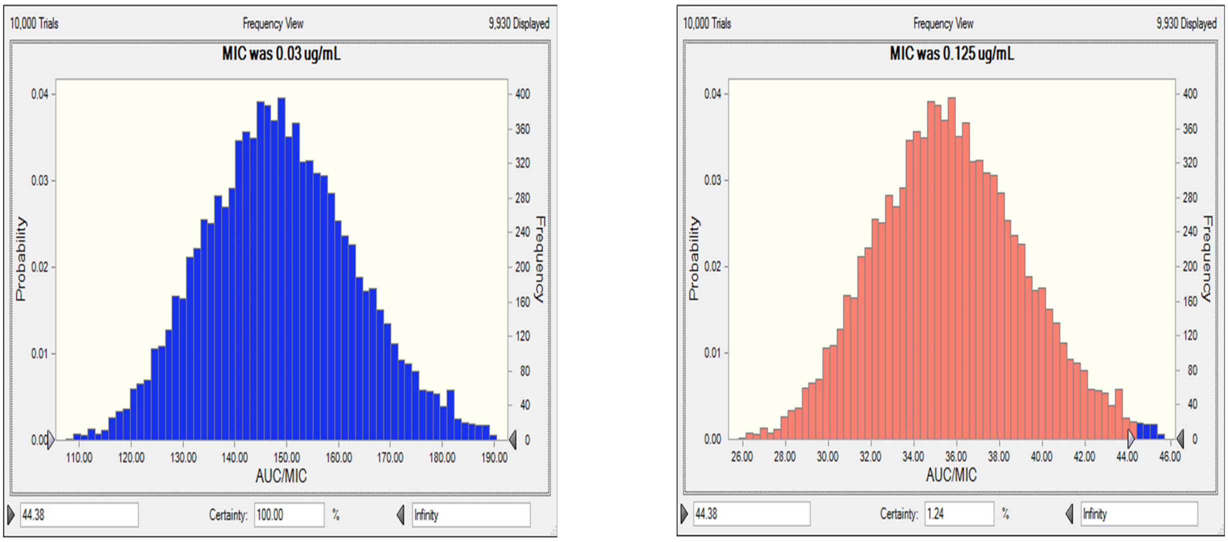

Figure S6. PTA of danofloxacin against *G. parasuis* in plasma.

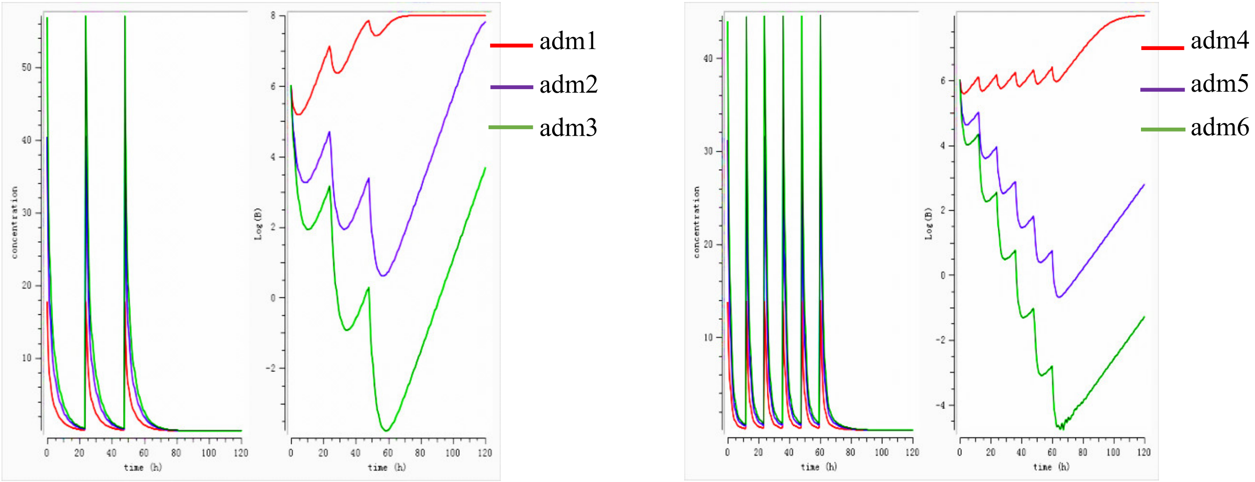

Figure S7. Forecast growth of *G. parasuis* at different dosage regimens. adm 1: prevent dosage: 4.58 mg/kg once daily; adm 2: therapeutic dosage: 10.32 mg/kg once daily; adm 3: eradicate dosage: 15.97 mg/kg once daily; adm 4: prevent dosage: 4.58 mg/kg twice daily; adm 5: therapeutic dosage: 10.32 mg/kg twice daily; adm 6: eradicate dosage: 15.97 mg/kg twice daily.

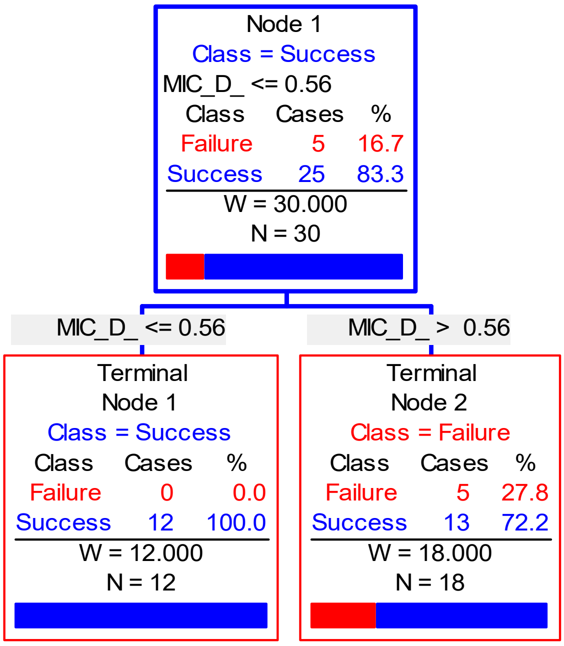

Figure S8. CART tree showing values of clinical outcome.

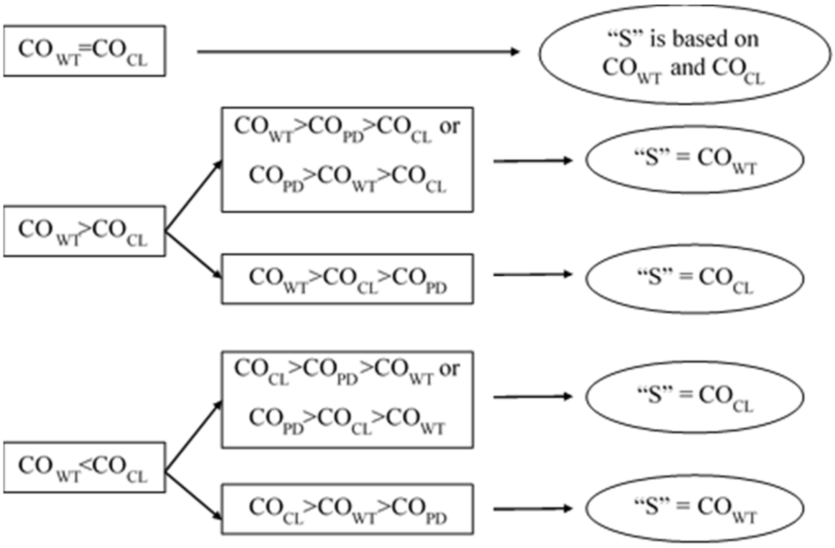

Figure S9. Susceptibility breakpoint decision tree.

Table S1. Epidemiological MIC for danofloxacin against *G. parasuis*.

Parameters	Value
MIC range	0.015 μg/mL–32 μg/mL
MIC_{50}	2 μg/mL
MIC_{90}	8 μg/mL
Selected Subset	≤64 μg/mL
Modal MIC	2 μg/mL
Log_2MIC Mode	1
Max Log_2MIC	6
Selected Log_2 Mean	1
Selected Log_2 SD	1
95.0% Subset ECOFFs	8 μg/mL

97.5% Subset ECOFFs	8 μg/mL
99.0% Subset ECOFFs	16 μg/mL
99.5% Subset ECOFFs	16 μg/mL
99.9% Subset ECOFFs	32 μg/mL

Selected Subset was the optimal fitting range by nonlinear regression; Modal MIC was the highest MIC distribution.

Table S2. Concentrations of danfloxacin in plasma and PELF at various time points (n = 6).

Time Points (h)	Plasma	PELF
0.08	0.59±0.04	
0.167	0.62±0.02	
0.25	0.67±0.02	
0.5	0.64±0.02	2.11±0.37
0.75	0.59±0.02	
1	0.52±0.04	3.13±0.35
1.5	0.47±0.05	3.89±0.11
2	0.41±0.04	3.51±0.33
3	0.35±0.04	3.02±0.21
4	0.30±0.05	2.23±0.25
6	0.24±0.05	1.56±0.45
8	0.17±0.03	1.02±0.23
10	0.12±0.02	0.69±0.19
12	0.09±0.01	0.38±0.16
24	0.07±0.02	0.27±0.03
36	ND	ND
48	ND	ND

"ND": not detected.
